# Analysis of Biogenic Amines and Small Molecule Metabolites in Human Diabetic Wound Ulcer Exudate

**DOI:** 10.1021/acsptsci.4c00418

**Published:** 2024-09-04

**Authors:** Lisa Gould, Morteza Mahmoudi

**Affiliations:** † Warren Alpert Medical School of Brown University, Providence, Rhode Island02912, United States; ‡ South Shore Health Center for Wound Healing, Weymouth, Massachusetts02189, United States; § Department of Radiology and Precision Health Program, Michigan State University, East Lansing, Michigan48824, United States

**Keywords:** chronic wounds, diabetic foot ulcers, wound exudate, metabolites, metformin, insulin, combined therapy

## Abstract

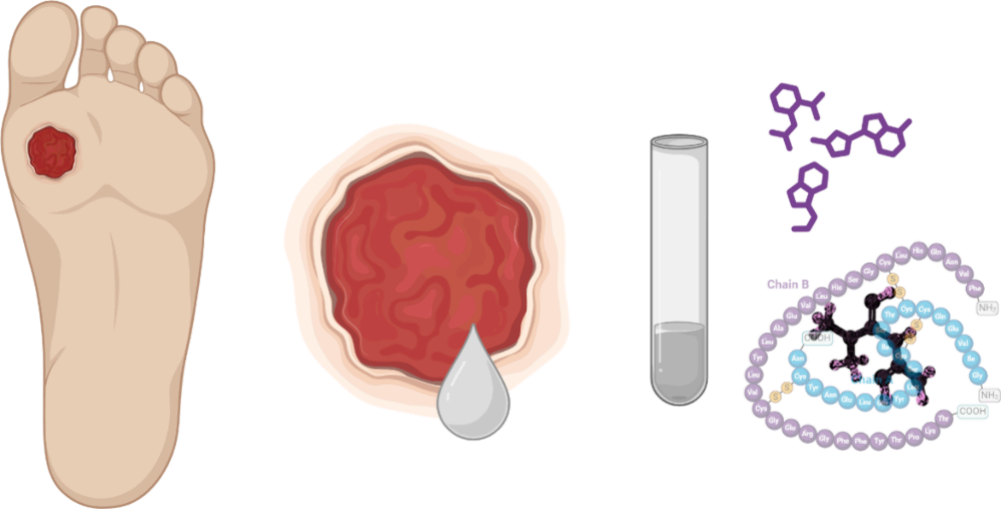

Diabetic foot ulcers (DFUs) pose a significant challenge in wound care due to their chronic nature and impaired healing processes. This study examines the biogenic amines and small molecule metabolites present in DFU wound exudates to identify their potential roles in wound healing. Under an IRB-approved protocol, wound fluid samples were collected from 25 diabetic patients and analyzed using ultrahigh-pressure liquid chromatography coupled with electrospray ionization quadrupole time-of-flight tandem mass spectrometry. The analysis identified 721 metabolites, with 402 confirmed through stringent criteria. Key metabolites significantly contributing to the wound exudates include betaine, lactic acid, carnitine, choline, creatine, and metformin (a widely used first-line treatment for type 2 diabetes). These molecules are known to influence wound healing processes, such as collagen synthesis, angiogenesis, inflammation modulation, and energy metabolism. Notably, the presence of drugs such as metformin and beclomethasone in the exudates suggests significant pharmacodynamic interactions that could influence wound healing. Specifically, we discovered that the combined use of insulin and metformin administered systemically significantly increased the concentration of metformin in the wound exudates (from 0.3% ± 0.0 to 3.1% ± 3.4; *p* = 0.00 49). This study highlights the complexity of DFU exudate composition and underscores the potential for targeted metabolic profiling to develop personalized wound care strategies.

Diabetic foot ulcers (DFUs) are a severe complication of diabetes, affecting a significant portion of the diabetic population and often leading to prolonged hospitalizations, amputations, and decreased quality of life.^1−3^ The chronic nature of DFUs is attributed to impaired wound-healing processes, which are influenced by a complex interplay of metabolic, inflammatory, and vascular factors.^1−3^ Despite advances in standard wound care, many DFUs fail to heal, necessitating a deeper understanding of the underlying mechanisms to develop more effective treatment strategies.^4^

Recent advancements in metabolomics have provided powerful tools for investigating the biochemical environment of various diseases.^5^ By analyzing the metabolites present in wound exudates, researchers can gain insights into the molecular processes that either promote or hinder wound healing. Biogenic amines and small molecule metabolites play pivotal roles in various physiological functions, including inflammation modulation, cellular energy metabolism, and tissue regeneration.^6−9^ Therefore, profiling these metabolites in DFU exudates could reveal crucial information about the wound microenvironment and identify potential biomarkers for therapeutic targeting. Although some studies have analyzed the proteomic and metabolomic composition analysis of DFU exudates for biomarker discovery,^10,11^ to the best of our knowledge, no prior research has conducted a comprehensive analysis of biogenic amines and small molecule metabolites in these exudates.

This study aims to analyze the biogenic amines and small molecule metabolites present in the exudates of DFUs using ultrahigh-pressure liquid chromatography (HILIC) coupled with electrospray ionization quadrupole time-of-flight tandem mass spectrometry (ESI QTOF MS/MS). By identifying and quantifying these metabolites, we seek to elucidate their roles in the wound-healing process and understand how factors, such as medication usage and metabolic states, influence their presence. Such detailed information about the metabolite composition of DFU exudates can provide valuable insights into the biochemical landscape of chronic wounds, offering potential pathways for developing personalized wound care strategies.

Through this comprehensive metabolic profiling, we highlight key metabolites that contribute to wound healing and explore the impact of pharmaceutical compounds such as metformin and beclomethasone on the wound environment. By correlating these metabolic profiles with clinical outcomes, our understanding of the biological significance of these metabolites would be enhanced and pave the way for innovative therapeutic approaches to improve healing in diabetic foot ulcers.

## Materials and Methods

### Human DFU Exudate Collection

Under an IRB-approved protocol, patients with Type 1 or 2 diabetes, between 18 and 80 years of age, with moderately draining ulcers below the level of the malleolus that failed to heal despite standard wound care, were identified. Following informed consent, wound fluid samples were obtained by placing sterilized, preweighed 1 cm^2^ Whatman glass fiber filters directly on the wound bed. The filter paper was allowed to be in contact with the wound until it was fully saturated. Triplicate samples were obtained and stored at −80 degrees until analysis. All samples were deidentified with subjects numbered 1–25. Health information included age, gender, smoking history, body mass index, most recent HgbA1c, diabetes medications, inflammatory markers, heart disease, recent corticosteroid use, and abnormal thyroid or immune function.

Exclusion criteria include active sepsis secondary to an infected foot ulcer, treatment with topical enzymatic debriding agents, current treatment with cellular/tissue products, and concurrent hyperbaric oxygen therapy.

### Analysis of Biogenic Amines and Other Small Molecule Metabolites By Ultrahigh-Pressure Liquid Chromatography (HILIC) Electrospray Ionization (ESI) Quadrupole Time-of-Flight Mass Spectrometer (QTOF) Tandem Mass Spectrometry (MS/MS)

The collected wound exudates were extracted from Whatman paper with lipids, proteins, and polar hydrophilic small molecules separated into various layers. Subsequently, biogenic amines and small molecule metabolites were analyzed using HILIC ESI QTOF MS/MS. Specifically, HILIC samples were injected using Agilent 1290 UHPLC coupled with a Sciex TripleTOF 6600 mass spectrometer. A short guard column protected the analytical UHPLC column, ensuring excellent retention and separation of various small molecule metabolites with narrow peak widths of 2–5 s and very good within-series retention time reproducibility, typically less than 1 s absolute deviation of retention times.

Chromatograms were analyzed for quality control, where internal standards were examined for consistency in peak height and retention time. Raw data files were processed using the latest version of MS-DIAL software, which identifies and aligns peaks and annotates them using both an in-house mzRT library and MS/MS spectral matching with the National Institute of Standards and Technology (NIST)/MoNA libraries. All MS/MS annotations were manually curated to ensure that only high-quality compound identifications were included in the final outcomes.

Data quantification was based on peak heights for the specific mz (mass-to-charge) value at the retention time (rt) value. Peak heights were preferred over peak areas due to their higher precision for low-abundance metabolites and less influence from baseline determinations. Additionally, overlapping ions or coeluting peaks are more challenging to deconvolute when determining peak areas compared to peak heights. Raw data peak heights were normalized to account for within-series drifts in instrument sensitivity, which can result from machine maintenance, aging, and tuning parameters. This normalization involved a variant of ″vector normalization”, where the sum of all peak heights for all identified internal standards for each sample (denoted as iTIC) was calculated. Peak heights for all samples were then normalized to the total average sum of the internal standards (iTIC).

## Results and Discussion

We analyzed the exudate compositions of biogenic amines and small molecule metabolites from human diabetic foot ulcers (*n* = 25). Our objective was to identify and quantify these molecules to understand their contributions and potential implications for wound healing processes. Our analysis detected 721 biogenic amines and small molecule metabolites. However, only 402 of these were detected with high confidence following the guidelines of the Metabolomics Standards Initiative (confirmed by matching at least the mass-to-charge ratio and retention time data). Consequently, our study focused on these 402 high-confidence compounds.

**Figure 1:**
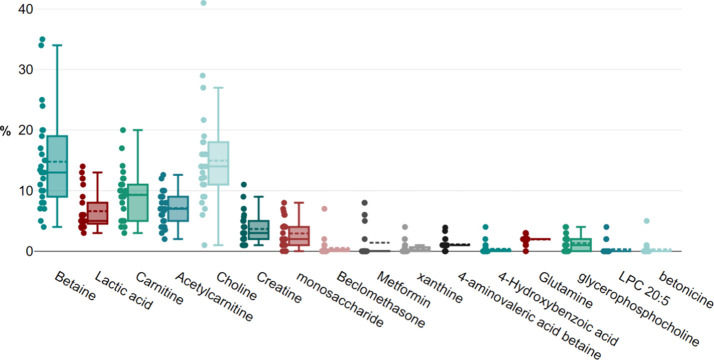
Box plot illustrating the percentages of the 16 key detected small molecules, each contributing over 3% in at least one of the exudates across all 25 sampled human cases. Each dotted line represents the percentage for an individual human exudate.

To determine the relative abundance of each small molecule, we calculated their relative contribution by dividing the peak height of each molecule by the sum of the peak heights of all detected molecules in each exudate and then multiplying by 100. From this analysis, we identified 16 small molecules that contributed at least 3% to one or more of the exudates. These 16 key molecules consist of betaine, lactic acid, carnitine, acetylcarnitine, choline, creatine, monosaccharide, beclomethasone, metformin, xanthine, 4-aminovaleric acid betaine, 4-hydroxybenzoic acid, glutamine, glycerophosphocholine, lysophosphatidylcholine (LPC) 20:5, and betonicine. The percentages of each of these small molecules in the 25 human exudates are summarized in Figure 1.

The key identified small molecules have useful functions in wound healing processes (see Table 1 for details). For example, betaine has multiple properties that positively affect wound healing, including osmoprotection, anti-inflammatory effects, enhanced collagen synthesis, promotion of angiogenesis, maintenance of osmotic balance, reduction of oxidative stress, and stimulation of key cellular activities.^12−15^ As another example, lactic acid promotes wound healing by encouraging angiogenesis, modulating inflammation, enhancing collagen synthesis, controlling infection, regulating pH, stimulating keratinocyte activity, maintaining moisture balance, and aiding in debridement.^16,17^ Carnitine is involved in energy metabolism, exhibits anti-inflammatory and antioxidant effects, promotes collagen synthesis and angiogenesis, improves cellular function, and modulates the immune response.^18,19^ These small molecules could collectively enhance wound healing by addressing various physiological and biochemical aspects that are crucial for effective tissue repair and regeneration.

**Table 1:** Potential Beneficial Functions of Identified Small Molecules in Wound Healing Processes

	positive properties on wound healing							
small molecule	improving skin cells’ function (e.g., migration and/or proliferation)	anti-inflammation	antioxidant properties	angiogenesis induction	infection control	regulation of pH	hydration/ moisture balance	improve debridement process
betaine	*	*	*	*			*	
lactic acid	*	*		*	*	*	*	*
carnitine	*	*	*	*				
acetylcarnitine	*	*	*	*				
choline	*	*		*				
creatine	*	*	*	*				
monosaccharide	*						*	
beclomethasone		*						
metformin	*	*	*	*				
xanthine	*		*					
4-aminovaleric acid betaine	*	*	*	*				
4-hydroxybenzoic acid	*	*	*		*			
glutamine	*				*			
glycerophosphocholine	*	*					*	
LPC 20:5	*			*				
betonicine	*	*					*	

One of the most striking observations from our study is that certain drugs, steroids, and supplements used by the patients, such as metformin, beclomethasone, and carnitine, were detected in the wound area. None of the identified molecules in the exudates were administered topically to the wound bed. Notably, some human exudates showed exceptionally high concentrations of these substances, which were considered outliers when examining all 25 human cases in the box plot presented in Figure 1. Given this unexpected finding, we conducted a more detailed analysis of the metformin data. Specifically, we investigated whether the combination therapy of metformin with insulin had a significant impact on metformin’s ability to reach the wound tissue. Our analysis revealed a noteworthy pattern: metformin’s contribution to the wound exudate is highly dependent on its coadministration with insulin. Although there are no direct pharmacokinetic interactions between insulin and metformin—meaning metformin does not affect the metabolism or elimination of insulin, and vice versa—our findings suggest that their pharmacodynamic interactions could be significant and warrant further detailed study.

**Figure 2:**
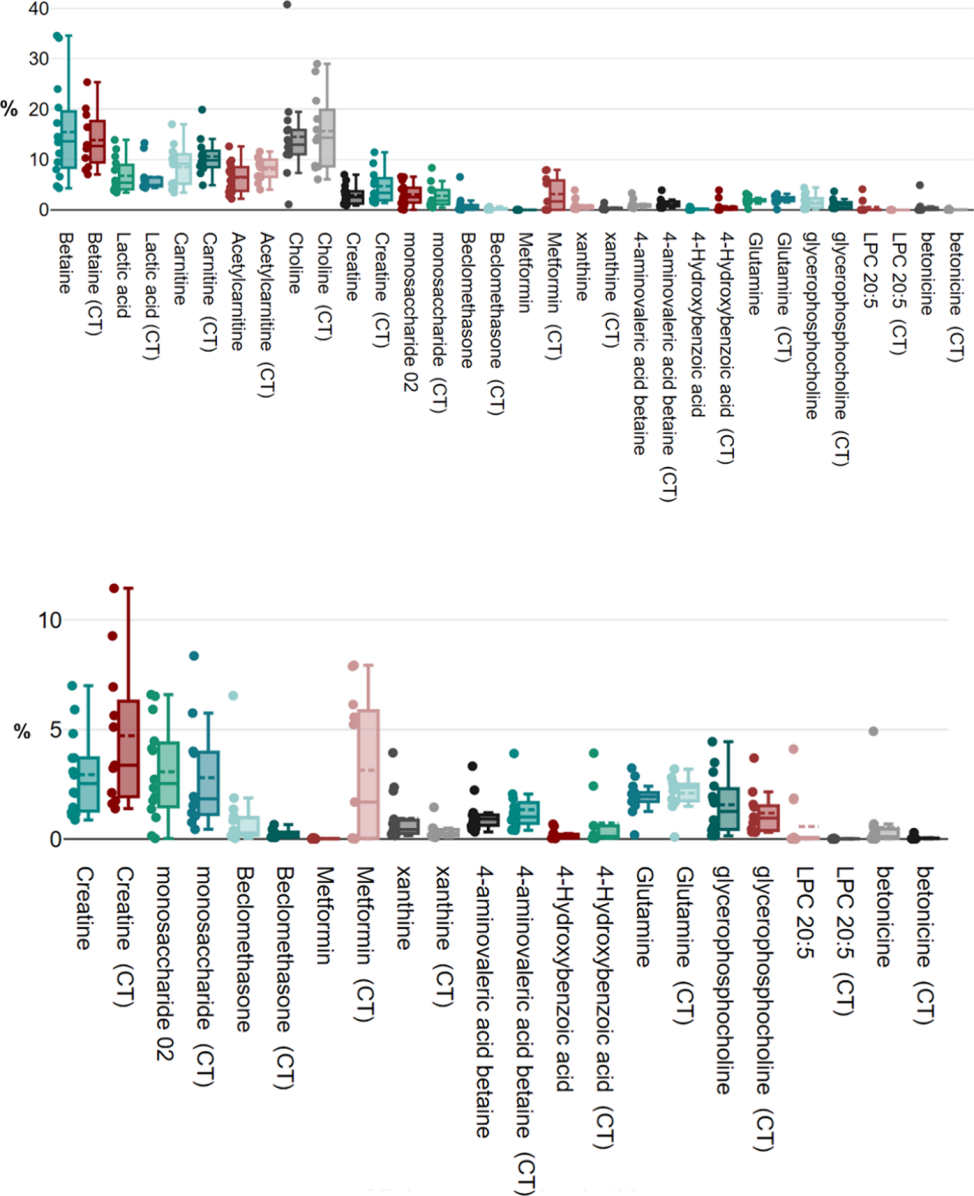
Box plot illustrating the comparison between the percentages of the 16 key detected small molecules in the combination therapy (CT) of insulin and Metformin versus non-CT categories. Each dot represents the percentage for an individual human exudate. The bottom box plot zooms in on small molecules with lower percentages in wound exudates, highlighting the critical role of CT in influencing Metformin concentrations in wound exudates.

Upon further examination, although some metformin concentrations were initially considered outliers in the box plot analysis (Figure 1), none of the highest metformin concentrations identified in the subsequent analysis (Figure 2) were deemed outliers. This suggests that the combination therapy of metformin and insulin facilitates a higher presence of metformin in wound tissues, indicating a potential interaction between these treatments that enhances metformin’s delivery or retention at the wound site. To further analyze the significance of this finding, we used one-way analysis of variance (ANOVA), which revealed significant differences between the combination therapy and noncombination therapy approaches (from 0.3% ± 0.0 to 3.1% ± 3.4; p = 0.0049). Additionally, we performed the Tukey HSD (“honestly significant difference”) test to confirm the 95% confidence intervals around the differences between the groups (diff = −2.8000, 95% CI = −4.6631 to −0.9369, *p* = 0.0049). This finding highlights the importance of considering the effects of combination therapies on drug distribution in the wound healing processes. Further research is needed to fully understand the mechanisms behind this interaction and its implications for optimizing wound care in patients receiving multiple medications.

The presence of metformin in wounds may substantially enhance the wound-healing process. In vitro studies suggest that metformin can promote faster and more effective tissue repair by directly affecting cells involved in wound healing, such as keratinocytes and fibroblasts.^20−23^ Additionally, metformin can reduce oxidative stress and inflammation,^24−26^ and promote angiogenesis,^27^ all of which contribute to improved wound healing outcomes.

The results presented in Figures 1 and 2 reveal significant interindividual variability in metabolite concentrations. These variations can be attributed to differences in metabolic states, dietary intake, medication usage, and overall health status of the individuals. Key findings include: (i) high variability in betaine and choline levels suggests differing dietary intake or metabolic processing among individuals; (ii) elevated lactic acid levels in certain cases indicate varying degrees of anaerobic metabolism, possibly due to differing physical activity levels or metabolic conditions; and (iii) the presence of pharmaceutical compounds like metformin and beclomethasone highlights the influence of medication on the metabolic profile of individuals.

In personalized medicine, the goal is to tailor medical treatment to the individual characteristics of each patient. The observed variability in key metabolites such as betaine, choline, and lactic acid and the presence of pharmaceutical compounds like metformin and beclomethasone suggest that each patient’s wound healing process is influenced by a unique set of biochemical factors. For instance, high variability in betaine and choline levels could be linked to differences in dietary intake or metabolic processing, indicating that nutritional interventions might need to be customized based on a patient’s specific metabolic profile. Similarly, elevated lactic acid levels could reflect varying degrees of anaerobic metabolism, pointing to the need for personalized physical activity recommendations or metabolic interventions.

The detection of pharmaceutical compounds in the wound exudates further highlights the influence of systemic medications on local wound environments. This suggests that the effectiveness of wound healing treatments could be significantly impacted by the patient’s medication regimen, necessitating adjustments in drug therapy to optimize wound healing outcomes. For example, the interaction between metformin and insulin, as observed in the study, could inform the development of combination therapies that enhance drug delivery and efficacy in wound care.

These insights into metabolite variability emphasize the potential of small molecule profiling to better understand individual health and disease states. By analyzing the specific metabolites present in a patient’s wound exudate, clinicians can gain valuable insights into the underlying mechanisms affecting wound healing. This knowledge can then be used to develop targeted therapies that address the specific needs of each patient, potentially improving the effectiveness of treatment and accelerating recovery.

Moreover, understanding the distribution and function of these metabolites in the context of wound healing can aid in the identification of biomarkers for disease progression and a therapeutic response. By correlating these metabolic profiles with clinical outcomes, future research can enhance our understanding of the biological significance of these metabolites, leading to the development of more precise and effective treatment protocols.

### Limitations of This Study

We acknowledge the potential limitations of this study, which is the relatively small sample size of 25 human DFU cases that may not fully capture the broader variability seen in a larger, more diverse population. Additionally, the study focused on the high-confidence detection of 402 biogenic amines and small molecule metabolites, but the exclusion of other metabolites that did not meet the stringent criteria could result in the loss of potentially valuable information. Another limitation is the cross-sectional nature of the study, which provides a snapshot of the metabolic environment at a single time point. This approach does not account for temporal changes in metabolite levels that could occur at different stages of wound healing. Furthermore, the study’s reliance on metabolomic analysis alone means that it does not integrate other biological data, such as genomic or proteomic information, which could provide a more comprehensive understanding of the wound healing process. Finally, while the study highlights the role of systemic medications, such as metformin and insulin, in influencing wound healing, it does not explore other potential confounding factors, such as variations in wound care practices or environmental influences, that could also impact the results. These limitations suggest the need for further research with larger cohorts, longitudinal study designs, and multiomics approaches to validate and expand upon the findings.

## Conclusions

This study provides a comprehensive analysis of the biogenic amines and small molecule metabolites present in the exudates of diabetic foot ulcers (DFUs), utilizing advanced mass spectrometry techniques to identify and quantify these crucial compounds. Our findings illustrate the complexity of the metabolic environment within DFUs, highlighting the significant roles of key metabolites, such as betaine, lactic acid, carnitine, choline, creatine, and metformin, in wound healing processes. These molecules influence critical functions, such as collagen synthesis, angiogenesis, inflammation modulation, and energy metabolism. The detection of pharmaceutical compounds such as metformin and beclomethasone in the wound exudates indicates important pharmacodynamic interactions that could impact wound healing. Notably, the combination therapy of insulin and metformin resulted in a significant increase in the concentration of metformin in the wound exudates. This observation suggests potential enhancements in drug delivery or retention at the wound site, indicating that combination therapies may play a crucial role in optimizing wound healing outcomes. The ability of insulin to potentially facilitate the localization and sustained presence of metformin in the wound environment underscores the importance of considering such synergistic effects in the design of treatment regimens for DFUs.

If the positive impact of combination therapies on wound healing is validated through larger clinical studies, this approach could revolutionize the management of nonhealing DFUs. Clinicians could leverage the synergistic effects of insulin and metformin not only to control blood glucose levels but also to enhance local wound healing processes. The combined use of these medications could improve drug bioavailability at the wound site, promote more effective tissue repair, and reduce the time required for wound closure. This strategy would have immediate clinical implications, enabling healthcare providers to prescribe both insulin and metformin as part of an integrated treatment plan for patients with nonhealing diabetic foot ulcers. Such an approach could lead to better patient outcomes, reduced complications, and potentially lower incidence of amputations associated with chronic DFUs. Additionally, this finding could encourage further research into other combination therapies that may similarly enhance drug delivery and therapeutic efficacy in wound care, paving the way for more personalized and effective treatment protocols for chronic wounds. Moreover, this knowledge has the potential to significantly enhance the therapeutic efficacy of current nanomedicine- and biomaterial-based wound dressings. By (i) understanding and analyzing the roles of wound exudates in shaping the biological identities of these advanced dressings^28^ and (ii) incorporating metformin and insulin into their formulations, we can potentially boost their healing properties. Such an approach could lead to more effective wound care solutions that are tailored to the unique metabolic and inflammatory environments of chronic wounds, ultimately improving patient outcomes and accelerating the healing process.

Our results reveal significant interindividual variability in metabolite concentrations, reflecting differences in metabolic states, dietary intake, medication usage, and overall health status among the patients. These insights emphasize the need for personalized approaches in wound care and the potential for small molecule profiling to inform targeted therapeutic strategies. Future research should focus on correlating these metabolic profiles with clinical outcomes to further elucidate their biological significance and optimize treatment protocols for DFUs. Understanding the intricate interplay of metabolites in wound exudates can pave the way for innovative treatments that enhance wound healing and improve patient outcomes, ultimately contributing to the better management of diabetic foot ulcers and other chronic wounds.
